# Clinical Competence and Perception of Medical Students after Early Clinical Exposure through Pre-clerckship Education at an Ethiopian Medical School: A Cross-sectional Study

**DOI:** 10.4314/ejhs.v32i6.19

**Published:** 2022-11

**Authors:** Esubalew Taddese Mindaye, Goytom Knfe Tesfay

**Affiliations:** 8 Department of Surgery, Saint Paul's Hospital Millennium Medical College, Addis Ababa, Ethiopia

**Keywords:** Early clinical exposure, pre-clerkship, medical education, Integrated curriculum

## Abstract

**Background:**

Conventional medical curriculum is the mainstay in the long history of modern medical education. Innovative integrated medical curriculum attracted significant attention in improving conventional curriculum. In the integrated curriculum, basic sciences are incorporated horizontally with each other, and students are exposed early to clinical settings. This is expected to improve students' knowledge and skills in clinical medicine by the time they start their clerkship rotation.

**Method:**

the study aims to make a baseline assessment on the overall knowledge and skills of medical students towards clinical medicine. An institution-based cross-sectional study was conducted from March to April of 2020 using 91 third year medical students (convenience sampling). A three-section self-administered survey instrument, short written MCQ exam, and practical (OSCE) students' examination were used for this survey.

**Result:**

participants tend to exhibit better knowledge on basics of history taking and physical examination with an average score of 79%. Comparatively, the score for average physical examination skill was low (56.3%). Students' perception on ECE showed, over 50% of participants believe ECE increases burden on their overall workload. Even then, the majority (92.3%) still think that ECE has positive impact on their clerkship education. Taken together, it appears more hands-on interventions is needed to further improve skills of medical students in physical examination with particular emphasis on the clinical examination of breast, thyroid, musculoskeletal, and neurologic systems.

## Introduction

Medical schools aspire to equip students with diverse skill set to care for physical, mental, social, and possibly spiritual well-being of their patients (1). Conventional medical education requires high level of streamlining because disjointed curriculum has been identified as one of the shortfalls for decline or stagnation in performance of graduates in delivering high-quality patient care(2,3). Specifically, there is a constant concern that graduates exhibit inadequate grasp of clinical skills, uncertain in their choices of diagnostic tests, lack of rational in prescribing pattern, decline in communication skills, and questionable ethical conduct (4).

In a typical conventional (Flexner) curriculum, students are taught first basic science of medicine in their pre-clerkship years (for 2- 3 years) and then followed by 4 weeks of physical diagnosis courses to bridge the forthcoming clinical year rotations (5). Thus, skills of physical diagnosis is important part of medical education that help trainee gain fundamental skills of clinical medicine and diagnosis (6). This approach may not necessarily meet the increasing demands of complexity and health needs of a growing population (2). In addition, students find preclinical subjects to be unattractive possibly due to theoretical nature of the course and lack of streamlining that made these courses less impactful in preparing students for clinical courses. In the Ethiopian medical school context, each preclinical department has its own scheduling and that is mostly not synchronized with other related or supportive courses from other departments (1). This asynchrony leads to unnecessary repetition of topics, loss of valuable time for all involved, confusion, and overall inefficiency (1). A new approach to teaching was needed and an integrated medical curriculum has been introduced into different medical schools. In this new approach, biomedical science courses are integrated horizontally with each other and vertically with clinical sciences so that students will be introduced about the basics of clinical medicine from early on in medical school. Interdependence in education is a key element as it underscores how various components interact with each other and it is considered necessary to provide inter-professional education that promotes collaborative practice (2). The major goal of integration is to reduce barriers between basic and clinical sciences and improve acquisition of skills through progressive development of concepts and their applications (7).

There are about 35 medical schools in Ethiopia that mainly use the conventional medical curriculum. As it is preferred by The Ministry of Health and Ministry of Education, there is a growing interest from most medical schools to adopt the integrated curriculum. In this regard, Saint Paul's Hospital Millennium Medical College (SPHMMC) is up front in introducing an integrated medical education curriculum in Ethiopia (introduced in 2010 G.C). To provide a baseline progress data to other aspiring institutions, we designed this study to assess the knowledge, skill, and attitude of medical students towards clinical medicine after completing their pre-clerkship study through the integrated medical curriculum. The findings of the study are expected to generate evidence for policymakers, input for the ongoing debate on how to teach medical education and provides information for further studies.

## Materials and Methods

**Study design**: The study was conducted at St. Paul's Hospital Millennium Medical College (SPHMMC), which is the second-largest multispecialty tertiary care teaching hospital in Ethiopia, Addis Ababa. The hospital has been giving medical services for more than seventy years with an emphasis on the underserved population. There are about 800 postgraduate and undergraduate Medical students, out of which 104 are year 3 medical students, and more than 3031 medical staff (8). An institution-based cross-sectional study was conducted from March 2, 2020 — April 15, 2020, G.C. Participants were drawn from all year 3 undergraduate medical students who have qualified to start their clerkship education. Dropouts and transferred students from other medical schools and those on sick leave were excluded.

**Sample size determination and sampling technique**: The sample size was calculated based on a prior presumption that the proportion (P) of students with adequate knowledge of clinical medicine would be about 50 % with the desired precision of (d = 0.05). The sample size was determined using the single population proportion formula and adding a nonresponse rate of 10%. Using convenience sampling, 91 subjects were included in the study. The variables assessed were basic clinical medicine knowledge, physical examination skill, and self-reported attitude and experience towards early clinical exposure.

Data collection and scoring: Data was collected using an MCQ short written exam to assess students' knowledge of basic history-taking and physical examination, and a self-administered semi-structured questionnaire was prepared to evaluate the impact of early clinical exposure in undergraduate medical education. Students' practical clinical examination skill was assessed by consultants and residents on volunteer medical students and interns with a checklist. The scope and structure of the questionnaire and the exam questions are included in Supplement 1. An objective assessment was conducted in regard to attitude, knowledge, and skill of medical students towards basic clinical medicine. Knowledge scores were calculated and summed up to give the total knowledge score (the maximum mark 20). The attitude and experience towards early clinical exposure statements were scored on a 5-Likert scale, from 1 to 5 (strongly agree 5; agree 4, neutral 3, disagree 2, and strongly disagree 1). Basic clinical examination skill scores were calculated and summed up to give a skill score out of 93. Participants were given sufficient time to read, comprehend, and answer all the questions, and information about the study was provided to address any questions.

**Data processing and analysis**: Data processing began with checking the data for accuracy and completeness. Each completed questionnaire was assigned a unique code and entered into Epi data (v3.1) and exported to SPSS version 26.00 (IBM, Armonk, NY, USA) for further analysis. Results are presented as frequency distributions, cross-tabulations, and graphs. Continuous variables are presented as mean and standard deviation (SD) and categorical variables as frequency and percentages. The chi-square test was used to evaluate the level of association. Operational definitions cut-off points used in this study are provided in the Supplement.

**Quality control**: Pretesting of the questionnaire was carried out using 5 randomly selected year 3 medical students. Following some input, the questionnaire was distributed to the study population.

**Ethical clearance**: Clearance was obtained from the Institutional Research and Ethics Review Committee (IRB) of the SPHMMC. Participation of the medical students in the study was voluntary and all data were kept confidential.

## Results

**Figure 1:**
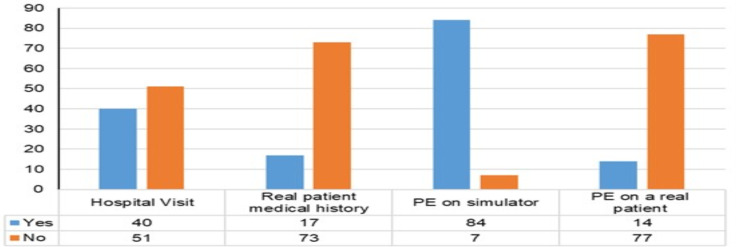
Early clinical exposure information

**Socio-demography**: With a response rate of 100%, 91 study subjects participated in the survey. The gender composition was 54.1% females and 45.9% males with an overall average age of 20.7 years (Table 1). Analysis of their responses revealed that most of the participants reported that they did not have any hospital visit yet and more of them have not seen any medical history taken or clinical examination performed on a real patient during their pre-clerkship clinical module (figure 1). Majority of the respondents reported they have attended physical examination on simulators.

**Table 1:** Socio-demography and proficiency of students on basics of history taking and physical examination

Socio-demography of study participants			
Characteristics	Category	Number	Percent (%)
Age(years)	18–20	35	38.8
	21–24	56	61.3
	Total	91	100
Sex	Male	42	45.9
	Female	49	54.1
	Total	91	100

Proficiency of students on basics of history-taking and physical examination (n = 91)		
Questions	Correctly answered (%)	Incorrectly answered (%)
Tactile fremitus finding of pleural effusion	36.3	63.7
Not respiratory system complaint	100	0
Not sign of respiratory distress	91.8	8.2
Percussion note of pneumothorax	85.9	14.1
Auscultation note of pleural effusion	64.8	35.2
Most lateral and distal heartbeat point	46.1	53.9
Heart sound by mitral valve closure	80.2	19.8
Not cardiovascular system complaint	98.9	1.1
Not character of jugular venous pressure	12.1	87.9
Not component of vital sign	97.8	2.2
Not important pre-requisite during abdominal examination	100	0
The main target of superficial abdominal palpation	96.7	3.3
Physical examination finding to differentiate splenomegaly from kidney mass	56.1	43.9
Not component of nervous system evaluation	98.9	1.1
Cranial nerve of tongue symmetry	93.4	6.6
Cranial nerve for hearing	98.8	1.2
Not component of motor system evaluation	65.9	34.1
Not urologic system complaint	98.9	1.2
Abdominal finding of ascites	94.5	5.5
Not component of history of present Illness	72.5	27.5

**Assessment of participants' knowledge of basic history-taking and physical examination**: Based on their performance on multiple-choice question (MCQ) evaluation, it appears majority of the students scored above 12 on a maximum 20 scale (60% passing mark). This implies that the majority appeared to have the required knowledge in examination or clinical medicine. The average knowledge score is 15.8 out of 20 (79%) with an SD of +1.5. Table 1 summarizes the proficiency of students in basic history-taking and physical examination. The results are sorted by specific area of examination. It appears, majority of the participants struggled to correctly respond to questions related to the characterization of Jugular venous pressure. Similar challenges have also been exhibited for questions related to tactile fremitus finding of pleural effusion, point of apical impulse and differentiating enlarged spleen from kidney mass on physical examination.

**Insight into the experience of participants with early clinical exposure during their pre-clerkship years**

As summarized in Table 2, the majority (98.9%) of participants responded good to excellent experience with ECE during pre-clerkship medical education. About 80% of the participants said ECE helped them with clinical terms and enhanced their self-motivation to attend clinics. However, more than half of the participants have not been to a hospital diagnostic environment yet because ECE is conducted by simulation and didactic lecture-based teaching methods at our college. Five out of ten participants believe ECE has increased their burden during pre-clerkship education but at the same time the majority (92.3%) thought that ECE has positive impact on their clerkship education. Of the total, 98.9% of the participants appear to be satisfied with ECE complementing their medical education (Table 2).

**Table 2:** Assessment of participants' experience with ECE during their pre-clerkship medical education (n=91)

Questions	Disagree	Neutral	Agree	SA
		
	(%)	(%)	(%)	
ECE creates interest in the Subject /topic	2.2	9.9	40.7	45
I had better topic understanding by the incorporation of ECE	3.3	17.6	51.6	27.5
Encouraged me to participate more in the teaching process	5.5	20.9	57.1	15.4
I found proper integration of Knowledge between basic & clinical science	12.1	20.9	50.5	12.1
Was more useful in providing relevant subject material	2.2	16.5	67	14.3
Ensured proper utilization of medical equipment like stethoscope	22	22	30.7	11
Causes better retention of topics than lecture-based classes	1.1	9.9	47.2	41.8
Helped me better recalling of topics	0	13.2	54.9	31.9
Helps Lifelong learning	1.1	12.1	59.3	27.5
Motivated me to study more on topics of basic science	11	35.2	35.2	16.5
Satisfied with guidance of teacher in ECE	18.7	22	44	12
I have become familiar with clinical terms	5.5	13.2	61.5	14.3
I have become familiar with hospital environment.	27.4	30.8	13.2	2.2
I like to learn with integrated curriculum in my future career	1.1	14.3	54.9	29.7
It helped me Score good marks	9.9	38.5	36.2	14.3
It increases confidence to deal with patients	7.7	28.6	50.5	12.1
It enhanced self-motivation to attend clinics	3.3	26.4	51.6	17.6
Does ECE increases course overburden on your pre-clerkship education	16.5	22	37.3	16.5
Do you think ECE have positive impact in clerkship education	0	6.6	56.1	36.2
Overall rating of ECE instruction method	Fair	Good	Very good	Excellent
	1.1	19.8	65.9	13.2

**Students' basic physical examination skills**: This survey showed only 48 students (52.7%) managed to score above the minimum passing mark of 60% on the overall physical examination skill. The overall average physical examination score of the participants was 56.3%, which is below the passing mark (Table 3). While majority of the participants gained some skills in the evaluation of pulse rate, respiratory rate, and body temperature but they have to refine other skills including hand hygiene during patient evaluation and effective use of BP apparatus. Most of the students have adequate skills in the evaluation of head, nose, eyes and ears, but nearly two-third of the participants did not demonstrate skills adequate for effective evaluation of breast and thyroid. Overall students who scored passing point on HEENT and lymphoglandular system were only 29% signifying more emphasis should be given in areas where students appear to score significantly below average.

**Table 3:** Summary of results for skills in evaluating vital signs, HEENT, and lymphoglandular system (n=91)

Essential physical examination skills checklist			Yes (%)	No (%)
Hand Hygiene		Washed with alcohol-based rub or 15 secs with soap and water	29.7	70.3
		Before touching the patients	29.7	70.3
		After finishing the exam	29.7	70.3
Vital sign measurement	Pulse rate measurement	Measured pulse rate by palpating radial pulse or auscultated at apex of heart at least 30 sec	93.4	6.6

	BP measurement	Used appropriate cuff size	65.9	34.1
		Placed on 2cm above the antecubital space	51.6	48.4
		Inflated cuff 30mmhg above pulse disappearance	35.2	64.8
		Deflated cuff 2–3mmhg per sec until 20 -30mmhg below the last sound	34.1	65.9

	Temperature	Measured and tell the normal temperature range (36.5 to 37.5)	78	22
	Respiratory rate	Measured Respiratory Rate- at least 30 sec	91.2	8.8

**HEENT and lymphoglandular system checklist**				
Head, Nose, Throat	Examined head/hair		95.6	4.4
	Performed inspection of nasal vaults		83.5	16.5
	Performed inspection of oral cavity		74.7	25.3

Eyes	Inspected conjunctiva (palpebral and bulbar)		89	11
	Checked pupillary response to light		49.5	50.5
	Correctly IDENTIFIES ophthalmoscope		45.1	54.9
	Examined External Ears		87.9	12.1
	Correctly IDENTIFIES otoscope to examine ears		50.5	49.5

Neck and lymphoglandular system	Palpated for lymph nodes, in all 9 areas		74.7	25.3
	Palpated the carotids at the level of thyroid cartilage		36.3	63.4
	Palpated the carotids right and left not at the same time		35.1	64.9
	Inspected thyroid from front or side of patient		51.6	48.4
	Palpated thyroid		45.1	54.9
	Examined the breast		34.1	65.9

Students' proficiency in examining respiratory and cardiovascular systems has been probed and the results are summarized in Table 4. Majority of participants (87%) appear to possess skills that is adequate for effective examination of the respiratory system. Similarly, the higher proportion of students demonstrated acceptable skill set in evaluating cardiovascular system with auscultation at aortic and pulmonary areas, but there is limitation in identifying positions of auscultation apart from upright position.

**Table 4:** Summary of results for skills in examining respiratory and cardiovascular systems (n =91) Essential physical examination skills

Essential physical examination skills			Yes (%)	No (%)
**Respiratory system Checklist**				
Inspection	Inspects- chest wall for shape and symmetry		96.7	3.3
	Cephalad to caudal (top to bottom)		93.4	6.6
Percussion	Bilaterally		78	22
	At least three areas (upper lobe to lower lobe)		79.6	20.4
Tactile fremitus	Upright, seated		96.7	3.3
	At two levels, at least (upper lobe and lower)		65.9	34.1
	Both right and left		86.8	13.2
Auscultation	Performed auscultation of posterior lung fields bilaterally upright, seated		84.6	15.4
	At least three areas (upper lobe to lower lobe) -both right and left		65.9	34.1
	Auscultates lateral lung fields -one area each, right and left		37.4	62.6
**Cardiovascular system checklist**				
Patient approach	Attends to patient comfort, through examination		89	11
Inspection	Precordium		96.7	3.3
	Neck veins, carotid pulse		69.2	30.8
	Apex of heart (Left lower sternal border, 5th intercostal space)		33	67
Palpation	At apex (apical impulse)		71.4	28.6
	Over right ventricle (Left Lower Sternal Border)		48.4	51.6
	At base (Right Upper Sternal Boarder, Left Upper Sternal Border)		10.9	89.1
Auscultation	Auscultation positions	Upright, seated	87.9	12.1
		Supine	49.5	50.5
		Left lateral	12.1	87.9
	Upper right sternal border (aortic area)		89.1	10.9
	Upper left sternal border (pulmonic area)		83.5	16.5
	Lower left sternal border (right ventricular area)		49.5	50.5
	Apical Impulse (Left ventricular area)		19.8	80.2
	Auscultates with both bell and diaphragm (all 4 areas)		48.4	51.6

Their scores in evaluating abdominal, musculoskeletal systems, and neurological disorders is summarized in Table 5. Majority of students (85.7%), with the exception for digital rectal examination, have demonstrated sufficient skills on abdominal examination. About 51.6% of the participants scored a passing mark on musculo-skeletal system evaluation. Except for motor system evaluation, majority of the participants demonstrated insufficient skills in the rest components of nervous system evaluation. Overall, only 41.9% of them scored passing mark on neurologic system evaluation.

**Table 5:** Summary of results for skills in examining abdominal, musculoskeletal systems, and neurological evaluation (n =91)

Essential physical examination skills		Yes (%)	No (%)
**Abdomen checklist**			
Demonstrated appropriate draping & exposure		94.5	5.5
Inspected abdomen		92.3	7.7
Auscultated abdomen for bowel sounds		93.4	6.6
Auscultates for abdominal bruits including aorta and bilateral renal arteries		78.0	22.0
Percussed Abdomen in 4 quadrants		87.9	12.1
Deep palpation, examined all 4 quadrants		89.0	11.0
Palpated liver edge		67.0	33.0
Percussed liver span at mid-clavicular line		71.4	28.6
Palpated spleen tip		59.3	40.7
Raised importance of doing Digital rectal examination		22.0	78.0
**Musculo-skeletal system**			
Palpated or percussed costovertebral angles for tenderness		48.4	51.6
Palpated spinous processes of thoracic and lumbar spine		60.4	39.6
Palpated paraspinal muscles of lumbar spine		54.9	45.1
Assessed spine range of motion	Flexion	78.0	22.0
	Extension	56.0	44.0
	Lateral bending	54.9	45.1
Extremity Examination	Inspects and palpates for deformities	89.0	11.0
	Palpates legs for edema with moderate pressure for 5 seconds	25.3	74.7
	Inspected feet for ulcers or deformities	61.6	38.4
Peripheral vascular examination	Palpates radial pulses, bilaterally	92.3	7.7
	Palpates brachial pulses, bilaterally	65.9	34.1
	Palpates posterior tibial pulses	54.9	45.1
	Palpates dorsalis pedis pulses	33.0	67.0
**Neurologic evaluation checklist**			
Motor evaluation	Isolates motor group, stabilizes across just one joint	78.0	22.0
	Clearly instructs patient on desired movement	56.1	43.9
	Provides moderate resistance to movement	67.0	33.0
	Shoulder abductors	65.9	34.1
	Elbow flexion, elbow extension	67.0	33.0
	Wrist flexion, wrist extension	63.7	36.3
	Hip flexion	82.4	17.6
	Knee extension, knee flexion	33.0	67.0
	Ankle dorsiflexion, ankle plantar flexion	53.9	46.1
	Great toe dorsiflexion	45.1	54.9
Reflexes	Selects/uses appropriate weighted reflex hammer	76.9	23.1
	Assesses arm DTR- biceps, triceps, brachioradialis	53.8	46.2
	Assess leg DTR- patellar reflex, achilles reflex	50.5	49.5
	Assesses Babinski response	36.3	63.7
	Normal ambulation	65.9	34.1
	Toe walk, heel walk	45.1	54.9
	Tandem walk	34.1	65.9
Sensory	Describe components of sensory examination	53.8	46.2
	Light touch in all extremities	50.5	49.5
Cerebellar function	Finger to nose	49.5	50.5
	Heel to shin	30.7	69.3

## Discussion

Equipping trainees with adequate knowledge and skills is key aim of medical schools to supply an increasingly demanding and complex healthcare system of a nation (9). Early clinical exposure tries to integrate the knowledge of basic and clinical sciences and create real context of medical practice (10). To achieve this goal, a well streamlined schedule that expose students with real clinical setup is required. In this regard, our survey appears to indicate there is still more work to be done to bring students into clinical settings as early as possible. More than half of the students did not have a single hospital visit or exposure to real patient in their 2 years of pre-clerkship education. They were trained mainly by simulation and lecture-based sessions. Students performed well on theoretical MCQ test give overall good credit to ECE in spite of their lower performance on the practical skill sessions. This may suggest the importance of engaging students in real patient evaluation and at the same time strengthening practical simulation practices. Attitude of participants in the current survey towards ECE was relatively high (71%). Further, the score for integration of basic and clinical science is about 62%, which appears to be lower than report by Mirzazadeh et. al. at 80.1% after ECE (10).

Only half of the participants were satisfied with the guidance they were getting from instructors during clinical module training. The clinical module is expected to be delivered by more experienced and pedagogically trained physicians, but in most instances junior and general practitioners with little pedagogic training are left to shoulder this burden. Part of the dissatisfaction of students might be attributable to this state of fact and this needs to be addressed. Nonetheless, the overall rating of participants with ECE was about 98.9% which was higher than those reported by Alka Rawekar et al. at 96.4% (9) or Mirzazadeh et.al. at 89.7% (10). Also, the majority of students in this survey (80%) reported ECE could be helpful even in their future continuing education preference, a finding comparable to reports of Alka Rawekar et al at 85% (9). Our study result showed ECE helps students acquire skills on clinical medicine similar to study done elsewhere (11).

In conclusion, it's encouraging that students get help in acquiring basic clinical knowledge in clinical medicine through pre-clerkship education, but it appears more work needs to be done to drastically improve the lower basic clinical skills. Hands on interventions might be helpful during their clerkship and clinical attachment with specific emphasis on clinical examination of the breast, thyroid, musculoskeletal and neurologic systems. The current survey showed there is a weakness in the implementation of integrated medical curriculum with ECE at SPHMMC. Exposing students to more clinical environment is needed and the ECE practice should be coached with more experienced academics. The role of simulation-based training as a bridge to real patient examination may be commendable but that should not replace the practical real-world teaching option. It is noteworthy that the integrated curriculum is demanding but it appears it has practical impact on student knowledge acquisition. Therefore, medical schools should give adequate attention and appropriately implement the integrated curriculum. This may mean that they require reorientation of faculties about the benefits of this curriculum. Furthermore, clinical departments should own the program and participate actively to help this program bear the desired fruit.

This is the first study to assess skills of medical students for basic clinical proficiency right after undergoing through ECE through integrated medical education curriculum at SPHMMC. The major limitation of the study was the fact that it is a cross-sectional study, and it is difficult to assess with such instrument the long-term impact of ECE on students' performance and attitude during the clinical years. Additionally, the study is limited by various assertions and definitive determination of cause-and-effect is very difficult. Furthermore, the data presented in the study are self-reported and the accuracy is partly dependent on the participants' honesty and ability to recall. Despite all these limitations, our findings provide a valuable insight into the knowledge, clinical skill and attitude of medical students towards basic clinical medicine after ECE.
